# Enhanced Photocatalytic H_2_ Generation by Light‐Induced Carbon Modification of TiO_2_ Nanotubes

**DOI:** 10.1002/open.202300185

**Published:** 2023-12-13

**Authors:** Amara Nasir, Alexander B. Tesler, Shiva Mohajernia, Shanshan Qin, Patrik Schmuki, Anca Mazare, Tariq Yasin

**Affiliations:** ^1^ Department of Materials Science and Engineering WW4-LKO Friedrich-Alexander University Erlangen-Nuremberg Martensstrasse 7 91058 Erlangen Germany; ^2^ Pakistan Institute of Engineering and Applied Sciences (PIEAS) PO Nilore Islamabad 45650 Pakistan.; ^3^ Chemical and Materials Engineering Department University of Alberta 12-237 Donadeo Innovation Centre For Engineering, 9211–116 St Edmonton Canada; ^4^ Regional Centre of Advanced Technologies and Materials Šlechtitel u 27 Olomouc 78371 Czech Republic

**Keywords:** annealing, TiO_2_ nanotubes, hydrogen evolution, organic electrolytes, photoelectrochemical water splitting

## Abstract

Titanium dioxide (TiO_2_) is the material of choice for photocatalytic and electrochemical applications owing to its outstanding physicochemical properties. However, its wide bandgap and relatively low conductivity limit its practical application. Modifying TiO_2_ with carbon species is a promising route to overcome these intrinsic complexities. In this work, we propose a facile method to modify TiO_2_ nanotubes (NTs) based on the remnant organic electrolyte retained inside the nanotubes after the anodization process, that is, without removing it by immersion in ethanol. Carbon‐modified TiO_2_ NTs (C‐TiO_2_ NTs) showed enhanced H_2_ evolution in photocatalysis under UV illumination in aqueous solutions. When the C‐TiO_2_ NTs were subjected to UV light illumination, the carbon underwent modification, resulting in higher measured photocurrents in the tube layers. After UV illumination, the IPCE of the C‐TiO_2_ NTs was 4.4‐fold higher than that of the carbon‐free TiO_2_ NTs.

## Graphical abstract

**A closer examination** of the role and impact of carbon within TiO_2_ nanotubes on H_2_ evolution and photoelectrochemical performance was carried out. Carbon is inherently present in nanotubes as remnant organic electrolyte used in the anodization processes. This residue serves as a carbon source during annealing in air, and when exposed to UV light, the carbon undergoes modification, thus resulting in enhanced photocatalytic efficiency.

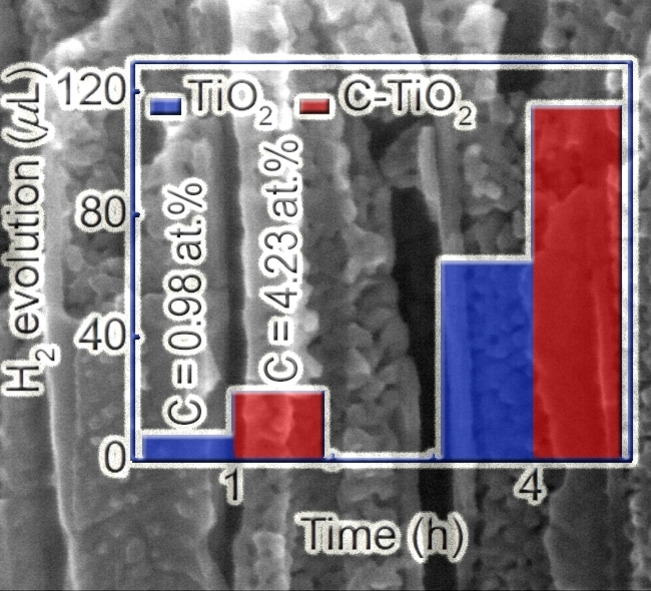

## Introduction

Among the available semiconducting materials, titanium dioxide (TiO_2_) is a material of choice for photocatalytic and electrochemical applications due to its outstanding physicochemical properties, such as chemical stability, low cost, and ample availability.[^1^, ^2^, ^3^, ^4^] While the position of TiO_2_ valence (VB) and conduction (CB) bands fit well with the photocatalytic splitting of water,[^1^, ^2^] its wide bandgap of 3.0–3.2 eV depending on the crystallographic phase (anatase or rutile), restricts its efficiency by limiting the light absorption to UV photons only.[^1^, ^2^]

Numerous approaches have been developed to overcome these intrinsic limitations. To shift the bandgap of TiO_2_ towards the visible spectral range and improve the lifetime of photogenerated charge carriers, bandgap engineering *via* doping with nonmetal elements,^[5]^ metals,^[6]^ and sensitization with organic dye molecules or quantum dots,^[7]^ has been employed. Conversely, 1D structuring of TiO_2_ surfaces has been used to maximize the specific surface area available for photocatalytic reactions and enhance its overall performance, and TiO_2_ nanotubes (NTs) have been investigated in a wide range of applications.[^1^, ^8^, ^9^] Such NTs combine the chemo‐physical features of TiO_2_, *e. g*., outstanding optical, electronic, and electrochemical properties, with an advantageous high‐surface area nanoscale geometry.[^1^, ^8^]

Carbon doping or modification with carbon materials has been attracting particular interest because the presence of carbon can lead to electron coupling between carbon and TiO_2_ or introduce localized occupied states that may narrow the bandgap of TiO_2_.^[10]^ Notably, nanotubular geometry bears advantages due to light management under light propagation along its longitudinal direction, and a direct charge transfer path can occur in an orthogonal direction.^[11]^ Classical carbon doping approaches of 1D TiO_2_ include annealing the TiO_2_ nanotubular layers in acetylene^[12]^ or acetylene/Ar mixtures.^[13]^ Typically, the surface carbon doping was confirmed by X‐ray photoelectron spectroscopy (XPS) or the presence of amorphous carbon by Raman spectroscopy.

Graphene oxide (GO) is one of the most promising carbon precursors for photocatalytic applications;^[14]^ however, fabricating such GO/TiO_2_ nanostructured materials is a complex process, often with scalability challenges. For instance, modifying TiO_2_ NTs with reduced GO is a multiple‐step process, showing good photocatalytic properties but with an inhomogeneous GO distribution on the NTs.^[15]^ Recently, a new approach for the synthesis of photoactive carbon‐modified TiO_2_ nanostructures was introduced by reducing graphene oxide (GO) on TiO_2_ photocatalysts under UV light illumination,[^16^, ^17^, ^18^, ^19^, ^20^] or oxidizing graphite on a defect‐engineered TiO_2_ photocatalyst^[21]^ opening up a new possibility to achieve photoactive composite materials.

The most commonly used method to incorporate carbon into TiO_2_ nanostructures is the addition of organic precursors, for example, chitosan,^[22]^ ethylene glycol (followed by vacuum annealing),^[23]^ glucose (followed by hydrothermal treatment)^[24]^ or polydopamine (followed by carbonization).^[25]^ More importantly, a feature of TiO_2_ NTs grown in organic electrolytes (ethylene glycol, glycerol, *etc*.) is their considerable carbon uptake from the electrolyte[^26^, ^27^] Therefore, this carbon may be directly utilized to obtain carbon‐modified TiO_2_ NTs, after annealing in reductive media (*e. g*., Ar).[^28^, ^29^] Carbon‐doping of anodic TiO_2_ layers as a result of the incorporation of organics from the electrolyte is intensively disputed, e. g., the case of TiO_2_ NTs obtained in organic electrolytes and annealed.[^30^, ^31^, ^32^]

Herein, we evaluate such high‐aspect‐ratio TiO_2_ nanotubes obtained by anodization in an organic‐based electrolyte and show that just by annealing in air, *i. e*., not in a reductive medium, the retained organic electrolyte results in amorphous carbon modification of the nanotubes. The air‐annealed C‐TiO_2_ NTs showed a two times higher H_2_ evolution amount than that of typical TiO_2_ NTs under UV illumination (without a sacrificial agent or co‐catalyst during the photocatalytic reaction). Notably, the UV‐illuminated C‐TiO_2_ NTs also revealed the modification of the incorporated carbon species (from the anodization electrolyte), and higher photocurrents (2.7‐and 4.4‐fold compared to C‐TiO_2_ NTs and TiO_2_ NTs, respectively) without the typical slow kinetics of the photocurrent transient encountered for TiO_2_ NTs.

## Results and Discussion

**Figure 1:**
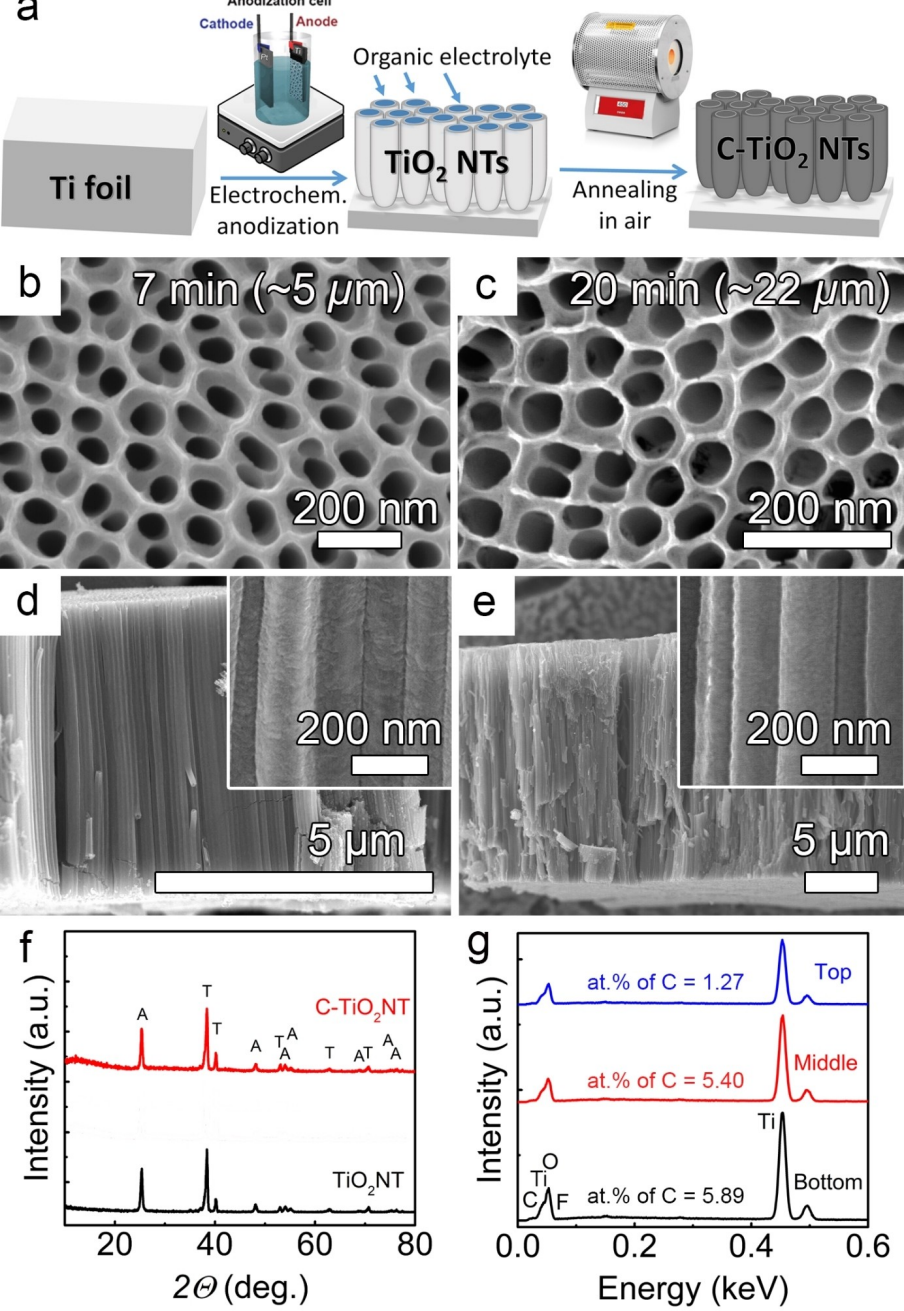
(**a**) Schematic representation of the C‐TiO_2_ NTs formation process. (**b**–**c**) Top‐view and (**d**–**e**) cross‐sectional HR‐SEM images of C‐TiO_2_ NTs. The inset images in (d–e) show higher magnification of the same NT images. (**f**) XRD spectra of annealed 10‐*μ*m‐thick samples (TiO_2_ and C‐TiO_2_). (**g**) EDX spectra at the top, middle, and bottom of 10‐*μ*m thick annealed C‐TiO_2_ NTs.

Figure 1a shows a schematic representation of the carbon modification process of the TiO_2_ NTs from anodization of the Ti foil to air annealing. First, anodic TiO_2_ NTs were grown in an organic‐based electrolyte of ethylene glycol, DI water, and 0.2 M NH_4_F at an applied potential of 70 V, using a two‐step anodization approach (as discussed in the experimental section) that leads to more uniform top morphology and tube length[^1^, ^33^] and Figure S1 in the Supporting Information. The NT length was controlled by the anodization time (5, 7, 10, and 20 min) resulting in NT lengths of 3.5, 5, 10, and 22 *μ*m – see selected top‐view and cross‐sectional SEM images in Figure 1b–e, for the 5‐ and 22‐*μ*m‐thick TiO_2_ NTs layers (the diameter of the NTs is of 139±16 nm for all thicknesses, as the applied potential was not changed).

**Figure 2:**
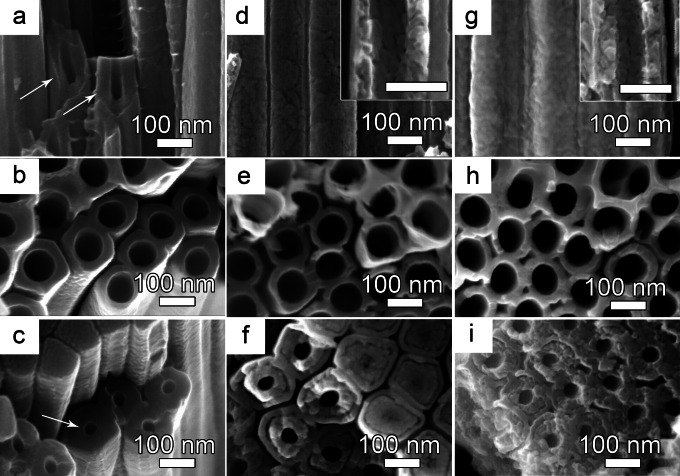
(**a**, **d**, **g**) Cross‐sectional, (**b**, **e**, **h**) top‐view, and (**c**, **f**, **i**) bottom‐view high‐resolution SEM images of (a–c) the as‐anodized TiO_2_, (d–f) annealed TiO_2_ NTs and (g–i) annealed C‐TiO_2_ NTs. Inset images in (d, g) are higher magnification of the annealed samples, the scale bar is 100 nm. The NT thickness was 10 *μ*m for all samples.

The NTs have a uniform, well‐ordered morphology with a typical V‐shape,[^1^, ^33^] which means that the tube diameter is higher at the top and decreases towards the bottom, with increasing tube wall thickness (as also seen in SEM images from close to top and bottom, Figure 2 a,b). Note that the anodized C‐TiO_2_ NTs were only briefly rinsed with DI water to retain the organic electrolyte within the NTs and use it as a carbon precursor, whereas the reference TiO_2_ NTs were immersed for 36 h in ethanol.

As‐anodized TiO_2_ NTs are amorphous;^[1]^ therefore, annealing was performed at 450 °C for 1 h in air to convert the layers to anatase (as the anatase phase is favored for many applications, and this annealing temperature leads to no cracks).^[34]^ The XRD spectra in Figure 1f exhibit sharp peaks at 2*θ*=25.3°, 48.1°, 54.0°, 55.1°, 62.7°, 68.8°, and 75.1°, confirming the anatase crystalline structure of the TiO_2_ and C‐TiO_2_ NTs (JCPDS card no. 21–1272),^[35]^ while the peaks at 2*θ*=38.6°, 40.2°, 53.1°, 63.0°, and 70.7° are indexed to the titanium substrate (JCPDS card no. 44–1294).^[36]^

We then evaluated the carbon content by EDX in both reference and C‐TiO_2_ NTs, the as‐anodized and annealed to anatase samples, and for the C‐TiO_2_ NTs also for 4 h UV (See Table S1 and S2 in the Supporting Information). The as‐anodized 10 μm‐thick C‐NTs had 11.8 at . % C and the annealing treatment decrease the C amount to ≈4.2 at . %. Whereas the bare TiO_2_ NTs had as‐anodized 5.4 at % C, and ≈1 at . % after annealing. Similarly, the fluorine in the F‐rich layer was burned off during annealing to ≈0.6–1 at . %. The distribution of C as a function of NT length was further analyzed by EDX for the 10‐*μ*m‐thick annealed C‐TiO_2_ NTs (Figure 1g) and indicated the presence of carbon all over the tube length (valid also for the as‐anodized layers), with a higher carbon content towards the tube bottom (where the inner layer of the NTs is thicker, see also Table S2 in the Supporting Information). This confirms that such a simple approach of using the intrinsic organic electrolyte within the NTs can be effectively utilized for the carbon modification of NTs without additional carbon sources.

It is worth clarifying a few critical aspects of anodic TiO_2_ nanotube morphology, namely that NTs obtained in organic electrolytes have a double‐walled NT structure,[^27^, ^37^] which is more easily seen upon annealing (the inner layer at the oxide/electrolyte interface is C‐rich because of the incorporation of organics, due to their decomposition, during the anodization process).[^38^, ^39^] This is why the as‐anodized TiO_2_ NTs show uniform, dense walls, and the double‐walled separation was not visible (Figure 2a–c). Annealing results in a porous inner shell due to the removal of C, and thus, the double‐wall structure is easily observed in SEM (more pronounced towards to tube bottom), for both annealed TiO_2_ NTs (Figures 2d–f and Figure S2 in the Supporting Information) and annealed C‐TiO_2_ NTs (Figure 2g–i). More importantly, comparing the tube walls of annealed reference and C‐TiO_2_ NTs, see Figure 2d, g, the reference TiO_2_ NTs show the typical clearly visible and separated grain boundaries along the tube walls,[^26^, ^40^] whereas the C‐TiO_2_ NTs show smaller, rougher grain boundaries and engraved^[40]^ tube walls.

**Figure 3:**
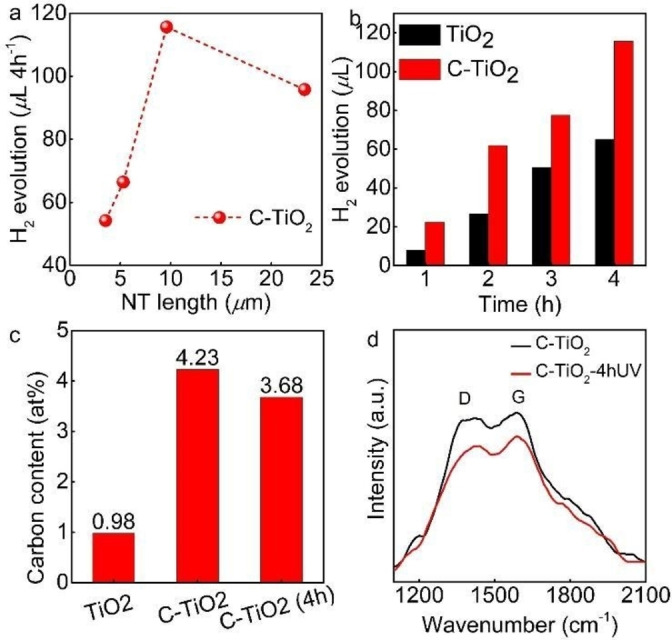
(**a**) H_2_ evolution of C‐TiO_2_ NTs with different NT lengths, (**b**) H_2_ evolution of 10‐*μ*m‐thick TiO_2_ and C‐TiO_2_ NTs in 0.1 M Na_2_SO_4_. (**c**) Comparison of carbon at . % content obtained by EDX analysis in 10‐*μ*m‐thick TiO_2_, C‐TiO_2_, and C‐TiO_2_‐4hUV NT samples. (**d**) Raman spectra of the 10‐*μ*m‐thick C−TiO_2_ NTs before and after 4 h UV light illumination.

In photocatalysis, the available surface area of the catalyst is exceptionally vital as it influences the photogenerated hot carriers′ capture and transfer and can be controlled by the TiO_2_ NT dimensions (length, diameter, and wall thickness). The H_2_ evolution of the obtained TiO_2_ and C‐TiO_2_ NTs of various thicknesses was evaluated by UV light illumination (*λ*=365±5 nm)^[41]^ for up to 4 h in 0.1 M Na_2_SO_4_ electrolyte. We first evaluated the influence of the C‐TiO_2_ NTs length on the H_2_ evolution amount (Figure 3a), with the highest amount measured for the 10‐*μ*m‐thick C‐TiO_2_ NTs. The optimal tube thickness for H_2_ evolution may be attributed to the interplay between morphology, *i. e*., NT length, and light absorption properties.[^42^, ^43^] The enhancement in the H_2_ evolution may be attributed to the increased surface area available for photocatalytic reactions due to the increase in NT length (since the diameter, wall thicknesses, and porosity are virtually similar in all the cases). At the same time, the reduction in H_2_ evolution for the 22‐*μ*m‐thick NTs may be attributed to pronounced light scattering phenomena.^[42]^ The optimal C‐TiO_2_ NT morphology, *i. e*., 10‐*μ*m‐thick NTs, was then evaluated in more detail, and a continuous increase in H_2_ evolution was observed (Figure 3b, and Figure S3 in the Supporting Information). Furthermore, a ~2‐fold enhancement in the H_2_ evolution after 4 h was observed for the C‐TiO_2_ NTs, compared to their TiO_2_ NT analogs.

**Figure 4:**
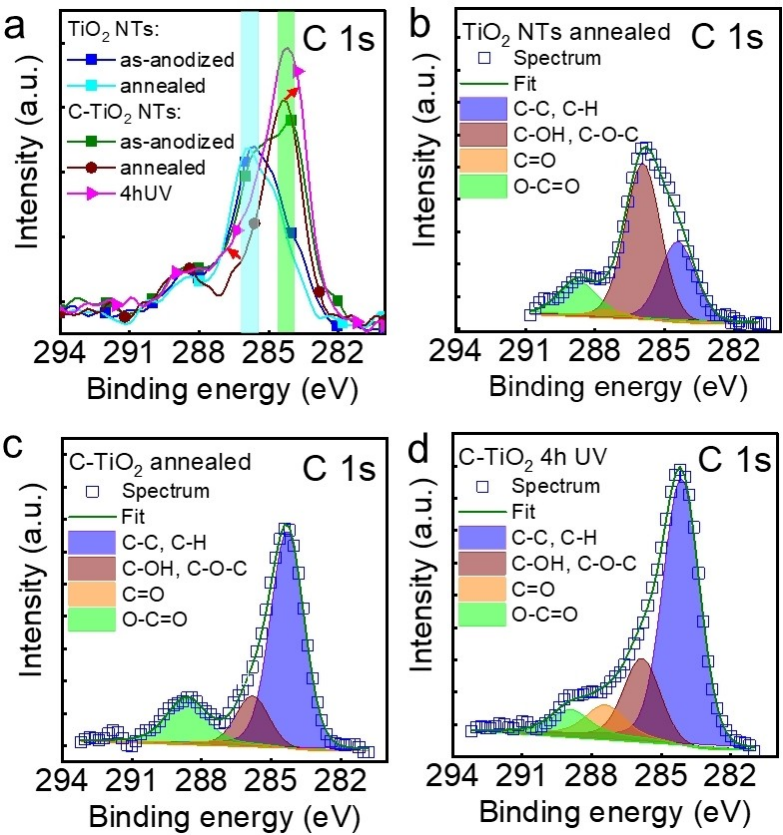
(**a**) High‐resolution C 1s XPS spectra of 10‐*μ*m‐thick TiO_2_ NTs and C‐TiO_2_ NTs as anodized, annealed, or for the latter 4 h UV. Experimental spectra and fitted high‐resolution XPS spectra of C 1s for (**b**) TiO_2_ NTs annealed and C‐TiO_2_ NTs annealed (**c**) before and (**d**) after 4 h UV light illumination.

Since the photoreduction of GO under UV light illumination was previously demonstrated,[^16^, ^17^, ^18^, ^19^, ^20^] in the next step we evaluated the content and nature of carbon before (C‐TiO_2_ NT) and after the 4 h UV illumination (C‐TiO_2_ NT‐4hUV). The EDX, Raman, and XPS spectroscopies results are summarized in Figures 3c–d, 4, and Figures S4–S6 in the Supporting Information. EDX measurements show that the as‐annealed C‐TiO_2_ NTs have a 4‐fold higher C content than their carbon‐free TiO_2_ NT analogs, which then decreases to 3.7 at . % C after UV light illumination but is still significantly higher (3.7 times) than the carbon‐free TiO_2_ NT (Figure 3c).

Raman spectra of the annealed C‐TiO_2_ NTs and C‐TiO_2_ NT‐4hUV (Figure S3 in the Supporting Information) show the bands corresponding to the typical modes of TiO_2_ anatase crystalline phase,^[44]^ *i. e*., E_g(1)_, B_1g(1)_, A_1g_+B_1g(2)_, and E_g(3)_, respectively. Carbonaceous materials have the G band associated with ideal sp^2^‐hybridized carbon systems (fully graphitized, *e. g*., graphene), while the D band is for disorder or sp^3^ hybridization (graphite).[^45^, ^46^] The as‐annealed and UV‐illuminated C‐TiO_2_ NTs samples also show in the Raman spectra (Figure 3d), the D band (1430 cm^−1^) and G band (1588 cm^−1^), which is similar to Raman spectra typically attributed to carbon‐based materials in literature, *e. g*., amorphous carbon[^29^, ^46^] or activated carbon materials.^[47]^ The slight decrease of both D and G bands upon UV illumination might indicate some loss of oxygenated carbon species and the increase of defects.[^19^, ^29^, ^48^, ^49^] XRD measurement of the UV exposed C‐TiO_2_ showed no difference (see Figure S5 in Supporting Information).

The XPS measurements confirmed the TiO_2_ structure (typical Ti 2p peaks of Ti^4+^, and O 1s of the metal oxide) with fluorine and carbon uptake from the electrolyte (C 1s – Figure 4a, and O 1s, Ti 2p and F 1s peaks – Figure S6 in the Supporting Information). From the high‐resolution C 1s peaks of the different TiO_2_ and C‐TiO_2_ NTs in Figure 4a, several key points are clear: *i*) as anodized TiO_2_ and C‐TiO_2_ show significant differences in the C 1s peaks with a higher peak intensity at ≈284 eV for the latter, *ii*) annealing does not influence the C 1s peak of the reference TiO_2_ NTs, but significantly alters that of C‐TiO_2_ NTs, and *iii*) the annealed C‐TiO_2_ NTs subjected to the 4 h UV illumination show a shift in the C 1s peak to slightly lower binding energies and a significantly broader peak at 286–288 eV.

Following, peak fitting the C 1s peak of the as‐anodized NTs (Figure S7) clarified the first point, confirming that the higher C−C, C−H peak at 284.2 eV (adventitious carbon,^[50]^ organic electrolyte)^[30]^ for the C‐TiO_2_ NTs is from the remnant electrolyte, while both samples have similar peaks attributed to C−OH, C−O−C bonds at 285.7 eV, C=O at 287.4 eV, and O−C=O at 288.8 eV.[^30^, ^50^, ^51^] With respect to the influence of annealing, the annealed bare TiO_2_ NTs show no significant difference in the C 1s peak compared to as‐anodized (Figure 4b and Figure S7a in the Supporting Information, though a decrease in the C=O peak is observed) – this is the result of the less carbon content in the reference NTs (removed by immersion in ethanol). Whereas the C‐TiO_2_ NTs show that with annealing, the C−OH, C−O−C peak at 285.7 and the C=O peak at 287.2 eV significantly decrease, while the C−C, C−H peak is slightly higher (Figure 4c). More importantly, after the 4 h UV illumination of the C‐TiO_2_ NTs, a shift with 0.2 eV to lower binding energies is observed for the C−C peak; in addition, higher C−C/C−H, C−OH/C−O−C, and C=O peaks indicate some rearrangement/modification of the carbon oxidized species. While no Ti−C peaks (≈281.5 eV)[^19^, ^52^] were observed in the XPS spectra, this means that the C is not doped in the TiO_2_ lattice (e. g., as it reported for C‐doped TiO_2_ nanostructures).^[53]^

The decrease in the O−C=O peak and increase in the C−O or C=O peak can be attributed to the reduction of oxidized carbon species, which after extended illumination time can also lead to degradation (e. g., degradation of GO on TiO_2_).^[19]^ Namely, when the carbon with oxygen functional groups is exposed to UV light in the presence of TiO_2_, it can undergo photodegradation, losing carbon (previous reports, in which graphene or GO on TiO_2_, subjected to UV illumination underwent first reduction and then photodegradation).[^19^, ^54^] A beneficial effect on kinetics (electron transport, electron‐hole recombination) and photocatalytic activity of the carbon‐containing TiO_2_ photocatalysts is achieved by the removal of the oxygen‐containing functional groups.[^55^, ^56^]

**Figure 5:**
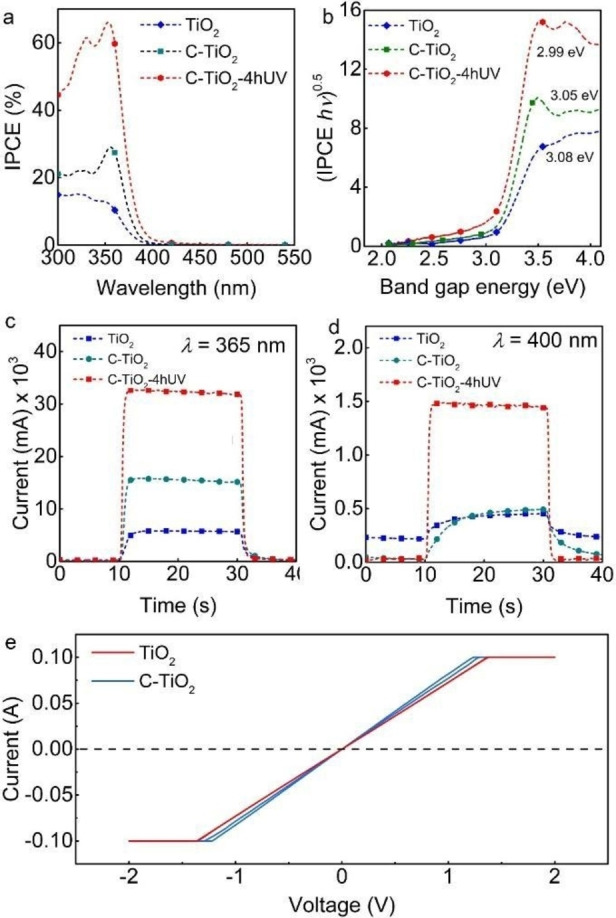
(**a**) IPCE efficiency, (**b**) bandgap calculations, and (**c**–**d**) current transients at *λ*=365 and 400 nm of TiO_2_ and C‐TiO_2_ NTs, and C‐TiO_2_NT‐4hUV. (**e**) Current–voltage (IV) curves obtained from the solid‐state conductivity measurements for TiO_2_ and C‐TiO_2_ nanotubes.

Figure 5 shows the incident photon to current efficiency (IPCE) spectra of the TiO_2_ NTs and the C‐TiO_2_ NTs before and after UV light illumination in 0.1 M Na_2_SO_4_ electrolyte. The highest IPCE value of 66 % at *λ*=353 nm was obtained with the C‐TiO_2_‐4hUV NTs. At the same time, the annealed C‐TiO_2_ NTs show an IPCE value of 28 % at *λ*=355 nm, while the carbon‐free TiO_2_ NTs show a maximum IPCE of 15 % at *λ*=322 nm. Thus, both evaluated C‐TiO_2_ NTs present a shift in the maximum IPCE value towards longer wavelengths. The bandgap of the TiO_2_ and C‐TiO_2_ NTs was obtained from the IPCE and diffuse reflectance optical measurements (Figures 5b and S8 in the Supporting Information). In both cases, the bandgap demonstrates a redshift towards longer wavelengths. The bandgap of anatase TiO_2_ NTs was estimated as 3.14 eV while shifting to 3.08 and 3.01 for C‐TiO_2_ and C‐TiO_2_‐4hUV NT layers, respectively. As a reference, we also measured the IPCE of the C‐TiO_2_ NTs immersed for 4 h in 0.1 M Na_2_SO_4_ but without UV illumination, *i. e*., kept in the dark (Figure S9 in the Supporting Information), and only a slight increase in the IPCE values relative to the C‐TiO_2_ NTs was observed, meaning that the C‐TiO_2_‐4hUV maintain their 2‐fold increase; thus, confirming that the enhancement in photocatalytic performance is due to effect of the UV treatment on the C‐TiO_2_ NTs.

Furthermore, C‐TiO_2_‐4hUV NTs demonstrate a slightly extended range of photocurrents towards the visible region and different photocurrent characteristics. The transient photocurrents were measured at *λ*=365 nm and 400 nm, and the 4 h UV illuminated C‐TiO_2_ NTs show a 5.5‐ and a 2‐fold improvement in photocurrents at *λ*=365 nm compared to C‐TiO_2_ and TiO_2_ NTs, respectively. This improvement may be attributed to better conductivity in the carbon‐enriched TiO_2_ layer.^[57]^ Additionally, a faster (no delay in the response time) and a more pronounced, 3‐fold improvement in photocurrent response was also observed at *λ*=400 nm compared to both the C‐TiO_2_ NTs and TiO_2_ NTs (Figure 5c–d). Whereas for C‐TiO_2_ and TiO_2_ NTs (400 nm), the slow transient rise kinetics typical of nanotube layers when switching on the illumination (due to trap filling in TiO_2_),[^56^, ^57^, ^58^] is also observed when switching off the illumination in a slow return. Such a delay in the response time is typical of annealed double‐walled NTs and can be attributed to numerous electron traps in the NTs, which lead to charge recombination.[^57^, ^58^, ^59^] Figure 5e shows the IV curves of the solid‐state conductivity measurements for annealed TiO_2_ and C‐TiO_2_ NTs, showing slightly higher currents for the latter.

In summary, we show that the carbon modification of the anodic TiO_2_ nanotubes can occur as a synergy between the remnant organic electrolyte and air annealing only, forming C‐TiO_2_ NTs, resulting in a higher H_2_ evolution amount upon UV illumination in aqueous solutions. Nevertheless, the remaining amount of carbon species from the organic electrolyte is crucial, as it determines their loading after the air annealing treatment. Furthermore, the UV illumination influences the resulting carbon by its modification, and as the C‐TiO_2_‐4hUV NTs show 13 % less carbon than C‐TiO_2_ NTs, meaning that it is not the carbon amount that influences the IPCE/photocurrent gradients, but the further effect of the UV illumination on the carbon in the nanotubes. This 13 % carbon amount decrease with 4 h UV illumination could be due to the reduction of the oxidized carbon species or a partial loss of carbon functional groups employed as a scavenger in the photocatalysis reaction. The effect of the carbon species modification is clearly evident in the substantial difference in IPCE, bandgap, and current transients for the C‐TiO_2_ before and after 4 h UV illumination.

## Conclusions

The present work shows that carbon‐modified TiO_2_ nanotubes can be prepared by a facile approach of annealing TiO_2_ NTs grown in organic electrolytes without any additional carbon source. This approach is based on the fact that the organic electrolyte is intrinsically present within NTs and can, therefore, be used as a carbon source for the annealing in air (while typically, this is achieved in literature by annealing C‐rich nanotubes in an Ar environment). The obtained C‐TiO_2_ NTs show the presence of carbon species and were investigated in photocatalytic H_2_ generation under UV illumination in aqueous solution, and a two‐times higher efficiency was obtained compared to bare TiO_2_ NTs. More interestingly, the UV illumination further influences the carbon modification, resulting in a 4.4‐fold higher IPCE efficiency and increased photocurrent responses, shifting the C‐TiO_2_ bandgap towards the visible spectral region. This study shows the potential of a simple platform for carbon modification of TiO_2_ nanostructures for enhanced H_2_ generation and photocurrents with a possibility of visible light activation. Further detailed and in situ studies would be necessary to fully investigate the nature of the carbon species present upon UV illumination.

## Experimental Section

### Preparation of Carbon‐enriched TiO_2_ Nanotubes (C‐TiO_2_ NTs)

TiO_2_ nanotubes (NTs) were grown on Ti foil (99.6 % purity, Advent, UK) in an organic electrolyte containing ethylene glycol (Carl Roth, Germany), 0.2 M ammonium fluoride (Carl Roth, Germany), and 2 M DI water (18.2 MΩ cm) at 70 V for 5, 7, 10, and 20 min at room temperature. The TiO_2_ NTs were prepared by two‐step anodization in a two‐electrode O‐ring cell. Ti foil with a 1 cm^2^ area was used as a working electrode, and Pt foil with a similar size was used as a counter electrode. The distance between the working and counter electrodes was set to 15 mm. After the first anodization step, the obtained TiO_2_ NTs were ultrasonically removed, and the substrate was further used in the second anodization step. After the second anodization step, the sample was gently rinsed with DI water to remove excess electrolyte, followed by annealing in air at 450 °C for 1 h. The reference sample was prepared similarly but was immersed in ethanol for 36 h to remove remnants of the organic electrolyte. Afterward, the reference sample was annealed at 450 °C for 1 h in air.

### Characterization Methods

A field‐emission scanning electron microscope (FE‐SEM, Hitachi, S4800, Japan) with energy‐dispersive X‐ray spectroscopy (EDX, EDAX Genesis, fitted to the SEM chamber) was used to characterize the morphology and chemical composition of the samples. The crystal phase structures of the samples were evaluated by X‐ray diffraction (XRD, X′pert Philips PMD diffractometer) with graphite monochromatized Cu irradiation (wavelength: *λ*=1.54056 Å) and Raman spectroscopy (LabRAM XoloRA, HORIBA JOBIN YVON SAS). The chemical composition of the samples was evaluated by X‐ray Photoelectron Spectroscopy (XPS, PHI5600, US), equipped with Al‐*K*_*α*_ monochromatic radiations (1486.6 eV). The spectra were calibrated to the Ti 2p at 458.5 eV. The background was subtracted using the Shirley method, and the peaks were fitted using the MultiPak software (Physical Electronics Inc., V9 A). Solid‐state conductivity measurements were performed using a 2‐point measurement setup composed of a USMCO micromanipulator and a precision semiconductor parameter analyzer (4156 C, Agilent technologies, Japan). The I−V curves were registered in the −2 to 2 V voltage window, using a sweep rate of 20 mV/s.

### Photochemical and Photoelectrochemical Characterizations

Photocatalytic H_2_ evolution was performed in 0.1 M Na_2_SO_4_ solution (pH=7). The illuminated area was approx. 1 cm^2^ for each sample. For photocatalytic H_2_ production at an open‐circuit voltage (OCP), the sample was immersed in a quartz reactor containing 0.1 M Na_2_SO_4_ electrolyte purged by N_2_ for 20 min before sealing. Afterward, the sample was irradiated with a polychromatic medium‐pressure Hg lamp (main peak at *λ*=365±5 nm,^[35]^ 30 mW cm^−2^) for 4 h. Photocatalytic H_2_ evolution was determined by gas chromatography (GCMS‐QO2010SE, SHIMADZU) with a thermal conductivity detector (TCD).

Photoelectrochemical properties were investigated using a three‐electrode cell consisting of Pt as a counter electrode, Ag/AgCl (3 M KCl) as a reference electrode, and an NT sample (either TiO_2_, C‐TiO_2_, or C‐TiO_2_‐4hUV) as a working electrode. The light source was a 150 W Xe lamp (Oriel 6365) with a monochromator (Oriel Cornerstone 7400 1/8 m). The current measurements were recorded by applying an external bias of +500 mV. The monochromatic light in the wavelength range of 300–600 nm with a 5 nm step size was used to evaluate the samples′ incident photon to current efficiency (IPCE). The transient photocurrents were recorded at *λ*=365 and 400 nm by chopped light chronoamperometry by illuminating each sample in the following sequence: (1) 10 s light off, (2) 20 s light on, and (3) 10 s of light off.

## Supporting Information

The supporting information consists of (1) HR‐SEM images of one‐step and two‐step anodization approach, (2) HR‐SEM images of the top, middle, and bottom of C‐TiO_2_ NTs, (3) The H_2_ evolution rate of 10‐*μ*m‐thick annealed TiO_2_ and C‐TiO_2_ NTs, (4) Raman spectra of 10‐*μ*m‐thick C‐TiO_2_ NTs and C‐TiO_2_NT‐4hUV, (5) XRD spectrum 10‐μm‐thick C‐TiO_2_NT‐4hUV, (6) XPS spectra of C 1s peak fitting of as‐formed C‐TiO_2_ NTs and C 1s, O 1s, and Ti 2p peaks of the as‐anodized, annealed, and UV illuminated C‐TiO_2_ NTs, (7) XPS spectra of C 1s peak fitting of the as‐formed TiO_2_ and C‐TiO_2_ NT (8) Kubelka‐Munk transformation of diffuse reflectance measurements of TiO_2_ and C‐TiO_2_ NTs, (9) IPCE efficiency of C‐TiO_2_ NTs immersed in 0.1 M NaSO_4_ for 4 h in dark and after UV illumination, (10) The atomic percentage (at . %) obtained from EDX measurements for the as‐anodized and annealed TiO_2_ NTs.

## Conflict of interests

The authors declare no conflict of interest.

1

## Supporting information

As a service to our authors and readers, this journal provides supporting information supplied by the authors. Such materials are peer reviewed and may be re‐organized for online delivery, but are not copy‐edited or typeset. Technical support issues arising from supporting information (other than missing files) should be addressed to the authors.

Supporting Information

## Data Availability

The data that support the findings of this study are available from the corresponding author upon reasonable request.
