# Supplementary material for: Enhanced Photocatalytic H2 Generation by Light‐Induced Carbon Modification of TiO2 Nanotubes
Source: ChemistryOpen. 2023 Dec 13;13(5):e202300185. doi: 10.1002/open.202300185 (PMC11095147; doi:10.1002/open.202300185)
Supplement: Supplementary file 1 — Supporting Information [file OPEN-13-e202300185-s001.pdf]

ChemistryOpen

Supporting Information

Enhanced Photocatalytic H_2 Generation by Light-Induced Carbon Modification of TiO_2 Nanotubes

Amara Nasir, Alexander B. Tesler, Shiva Mohajernia, Shanshan Qin, Patrik Schmuki,* Anca Mazare,* and Tariq Yasin

1. HR-SEM images of one-step and two-step anodization approach

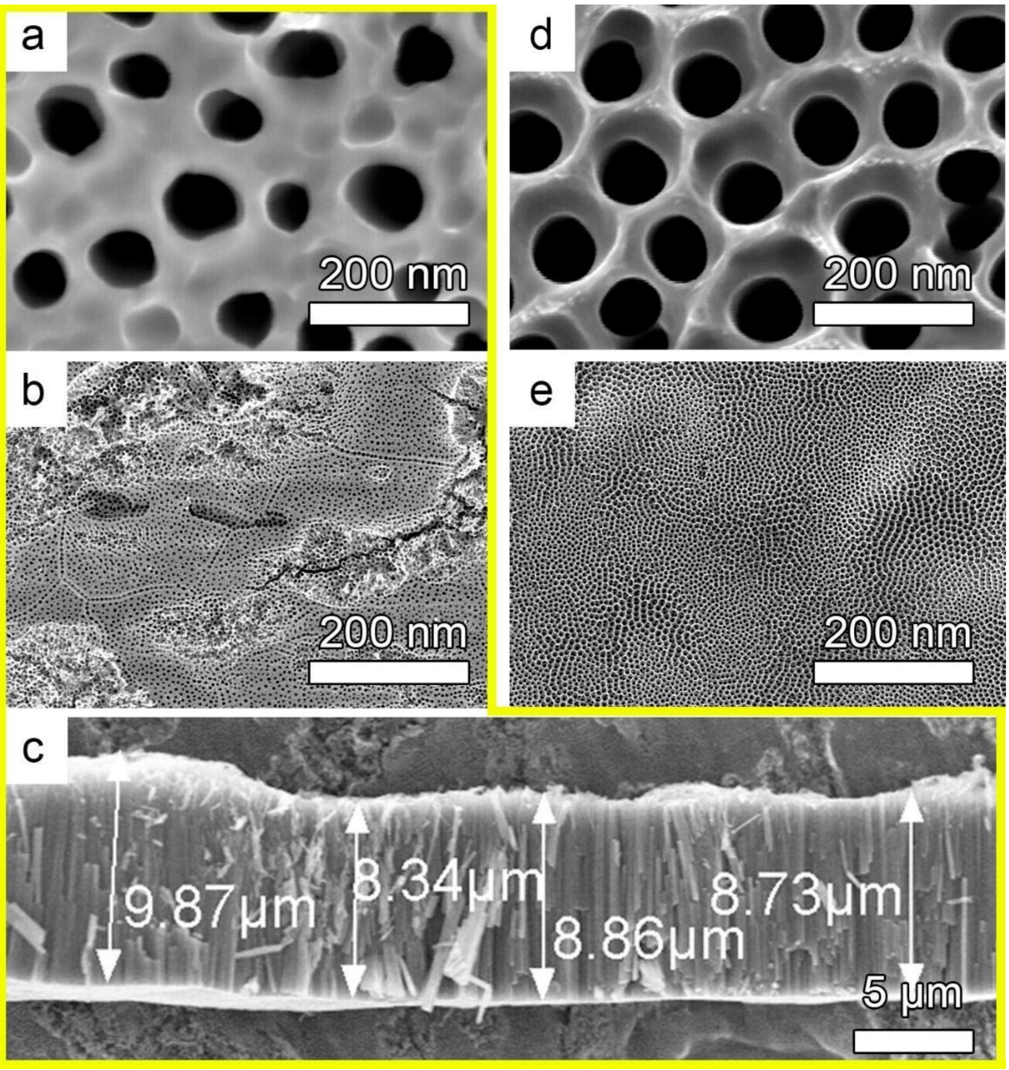

Figure S1. One-step vs. two-step anodization (70 V, 10 min: tube length of 10μm): Top-view FE-SEM images of (**a,b**) one-step anodization, and (**d,e**) two-step anodization (clearly showing the improved order degree for the two-step anodization), and (**c**) cross-sectional FE-SEM images of the one-step anodization layer showing the variation in tube length.

2. HR-SEM images of the top, middle, and bottom of C-TiO_2 NTs.

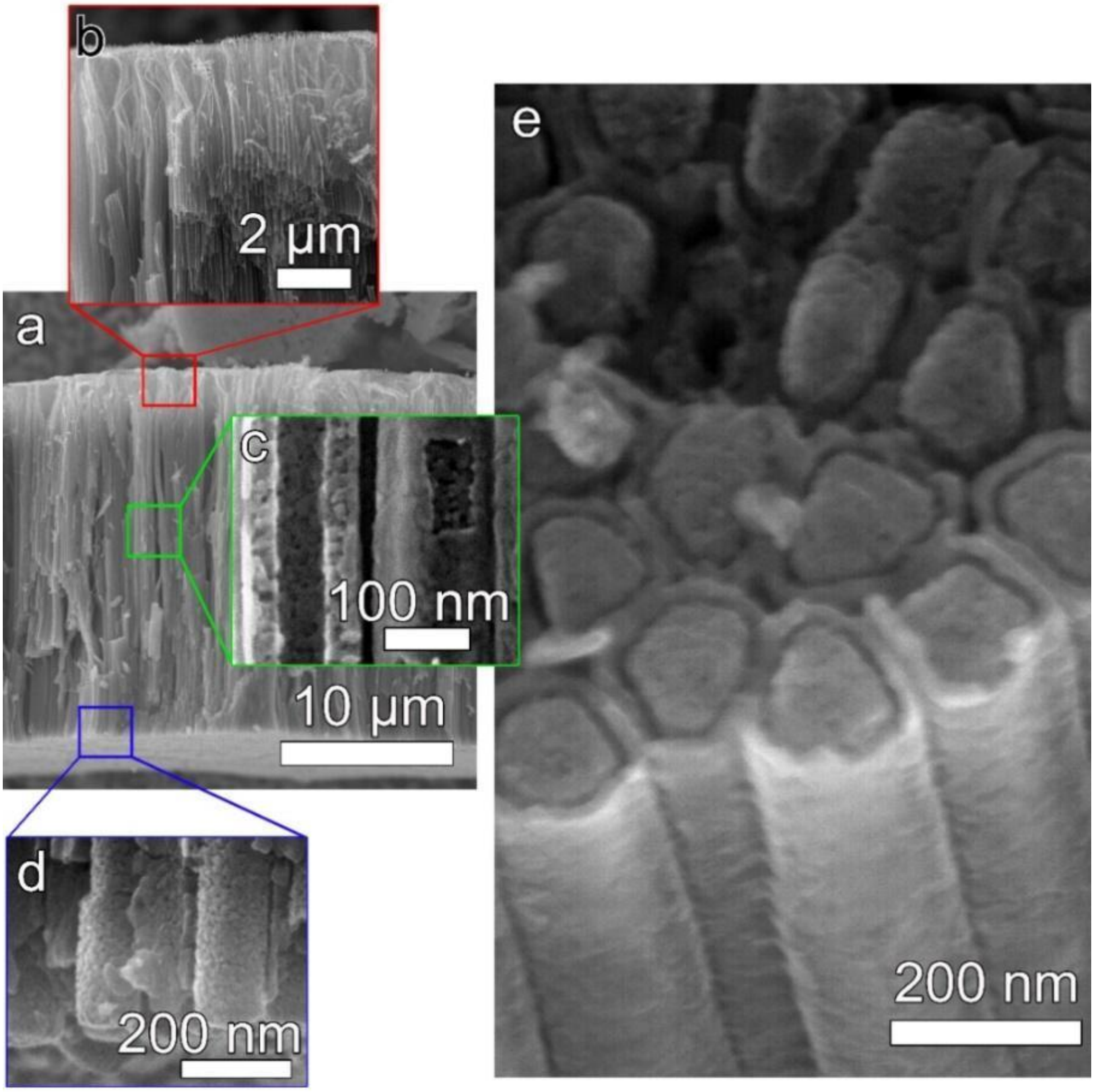

Figure S2. Cross-sectional FE-SEM images of 22-μm-thick annealed C-TiO_2 NTs of (**a**) full cross-section, (**b**) top, (c) middle, and (**d**) bottom parts of the C-TiO_2 NTs. (**e**) The bottom part of 22-μm-thick annealed C-TiO_2 NTs demonstrates a double-walled structure, where the inner core is porous, and the outer shell is dense.

3. The H_2 evolution rate of 10-μm-thick annealed TiO_2 and C-TiO_2 NTs.

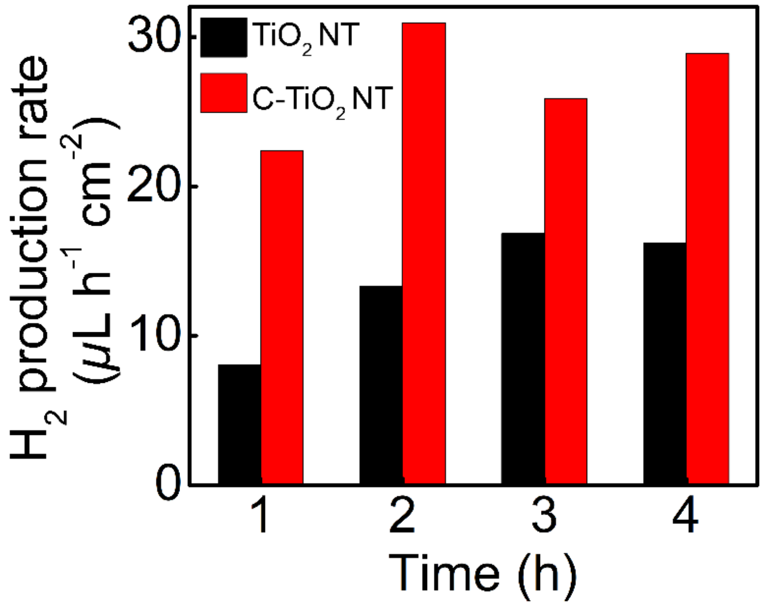

Figure S3. The H_2 evolution rate of 10-μm-thick annealed TiO_2 and C-TiO_2 NTs.

4. Raman spectra of 10-μm-thick C-TiO_2 NTs and C-TiO_2NT-4hUV.

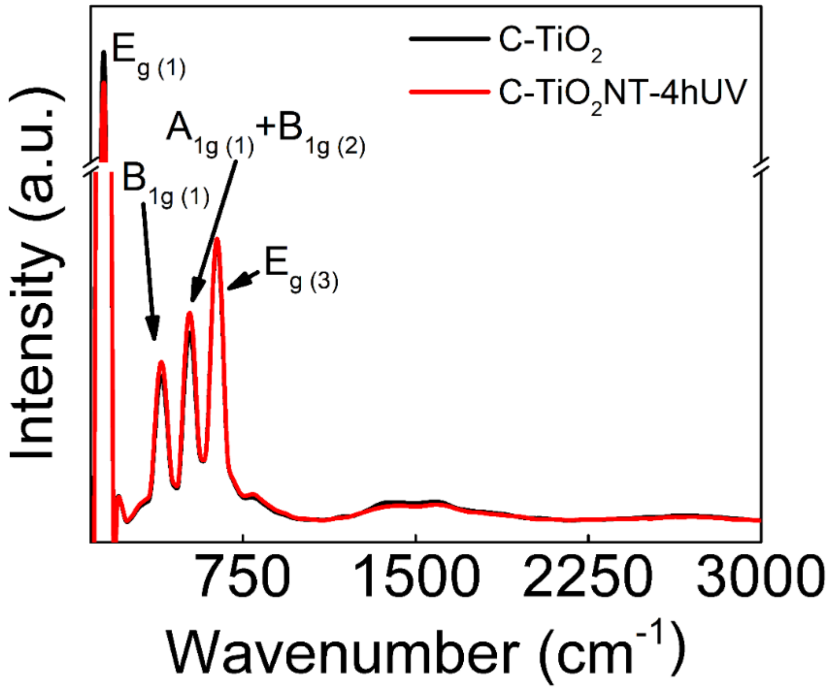

Figure S4. Raman spectra of 10-μm-thick C-TiO_2 NTs and C-TiO_2NT-4hUV.

5. XRD spectrum 10-µm-thick C-TiO_2NT-4hUV

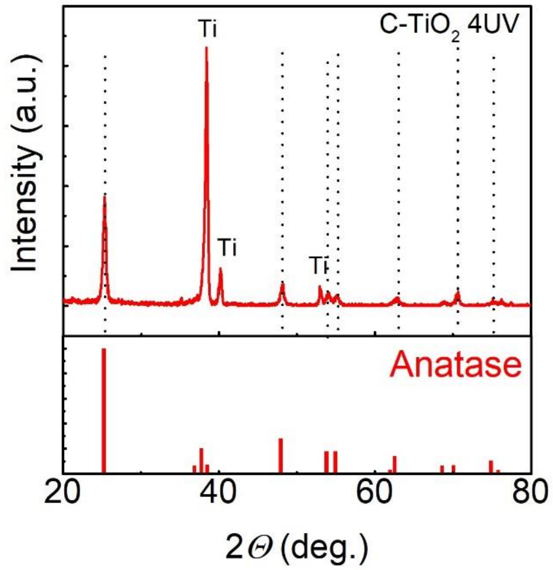

Figure S5. XRD spectrum of 10-*µ*m-thick C-TiO_2NT-4hUV.

6. XPS spectra of C 1s peak fitting of as-formed C-TiO_2 NTs and C 1s, O 1s, and Ti 2p peaks of the as-anodized, annealed, and UV illuminated C-TiO_2 NTs.

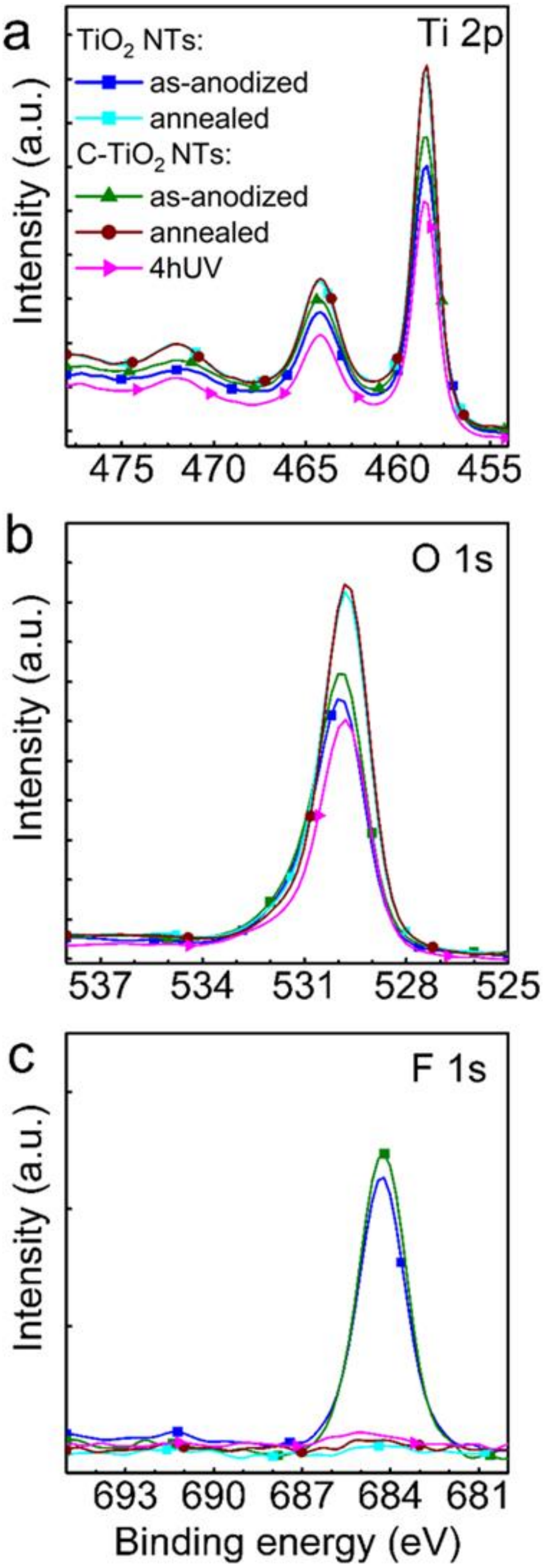

Figure S6. XPS spectra of (**a**) Ti 2p, (**c**) O 1s, and (**d**) F 1s peaks of the as-anodized, annealed, and UV-illuminated C-TiO_2 NTs.

7. XPS spectra of C 1s peak fitting of the as-formedTiO2 and C-TiO2 NTs.

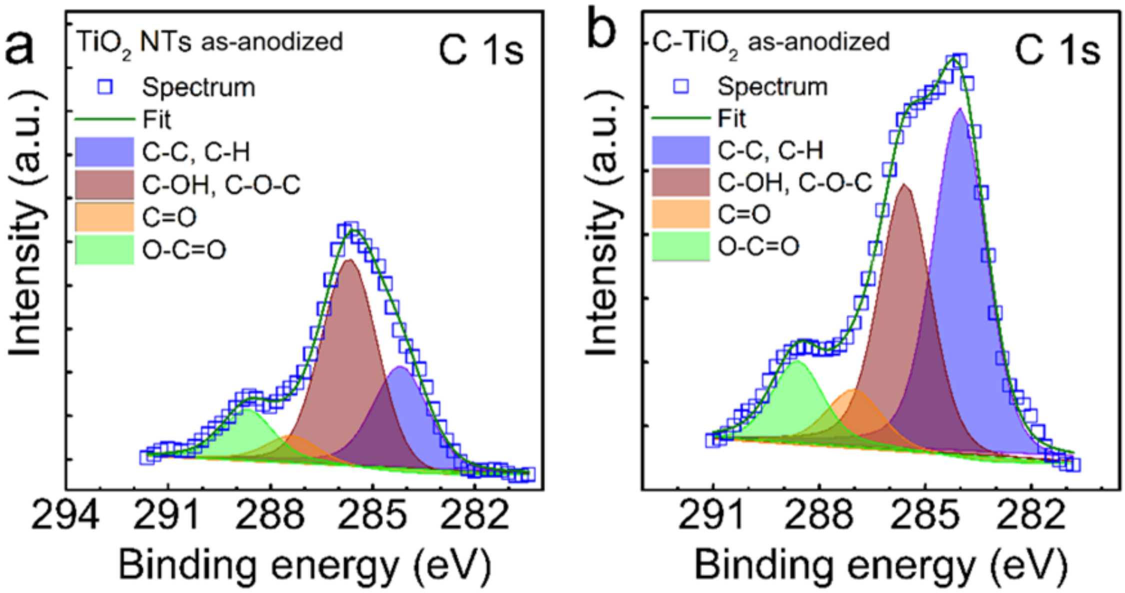

Figure S7. XPS spectrum of C 1s peak measured and fitting of the as-anodized (**a**) TiO_2 and (**b**) C-TiO_2 NTs.

8. Kubelka-Munk transformation of diffuse reflectance measurements of TiO2 and C-TiO2 NTs.

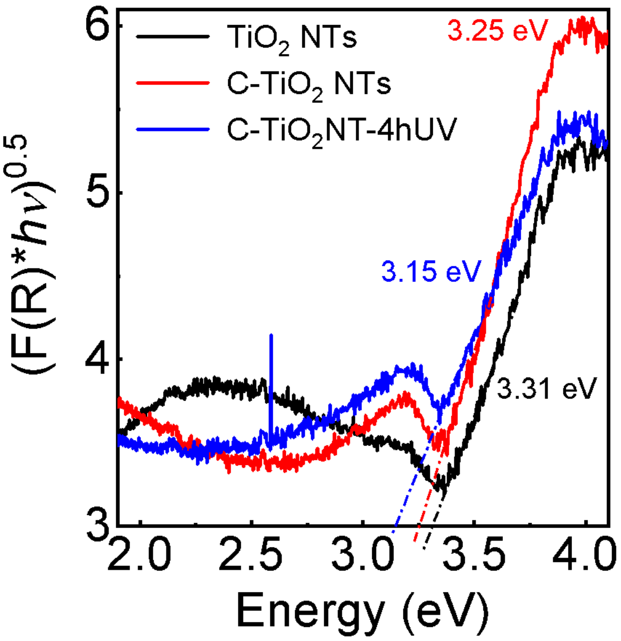

Figure S8. Kubelka-Munk transformation of diffuse reflectance optical measurements for indirect TiO_2 semiconductor.

9. IPCE efficiency of C-TiO_2 NTs immersed in 0.1 M $NaSO_4$ for 4 h in dark and after UV illumination.

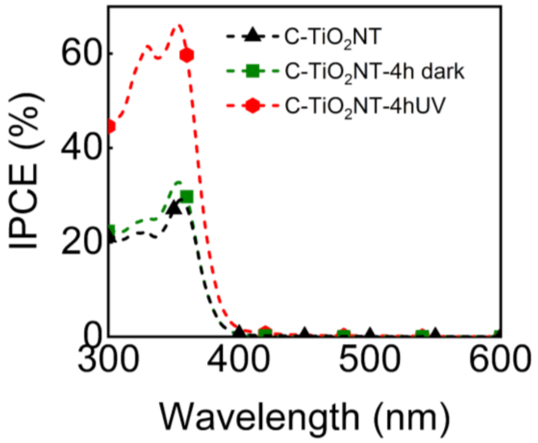

Figure S9. IPCE efficiency of C-TiO_2 NTs immersed in 0.1 M $NaSO_4$ for 4 h in the dark and after UV illumination.

10. The atomic percentage (at.%) obtained from EDX measurements for the as-anodized and annealed TiO_2 NTs.

Table S1. The atomic percentage (at.%) obtained from EDX measurements for the as-anodized and annealed 10-μm-thick TiO_2 NTs. The measurements were obtained at top-view configuration.

Sample	**C**	**O**	**F**	**Ti**
**As-anodized TiO_2 NTs (at.%)**	5.45	47.80	5.77	40.98
**As-anodized C-TiO_2 NTs (at.%)**	11.84	42.80	5.64	39.72
**Annealed TiO_2 NTs (at.%)**	0.98	54.19	1.06	43.77
**Annealed C-TiO_2 NTs (at.%)**	4.23	45.57	0.64	49.56
**C-TiO_2 NTs-4h UV (at.%)**	3.68	51.55	0.36	44.40

Table S2. The atomic percentage (at.%) obtained from EDX measurements for the as-anodized and annealed 10-μm-thick C-TiO_2 NTs as a function of NT length.

**Element**	**As-anodized C-TiO_2 NTs (at.%)**			**Annealed C-TiO_2 NTs (at.%)**		
	**Top**	**Middle**	**Bottom**	**Top**	**Middle**	**Bottom**
**Carbon**	12.47	11.49	10.76	1.27	5.40	5.89
